# The dynamic role of autophagy and MAPK signaling in determining cell fate under cisplatin stress in osteosarcoma cells

**DOI:** 10.1371/journal.pone.0179203

**Published:** 2017-06-09

**Authors:** Sudeshna Mukherjee, Subhra Dash, K. Lohitesh, Rajdeep Chowdhury

**Affiliations:** Department of Biological Sciences, Birla Institute of Technology and Science (BITS), Pilani, Rajasthan, India; Univerzitet u Beogradu, SERBIA

## Abstract

Osteosarcoma (OS) is an aggressive bone malignancy commonly observed in children and adolescents. Sub-optimal therapy for years has irretrievably compromised the chances of OS patient survival; also, lack of extensive research on this rare disease has hindered therapeutic development. Cisplatin, a common anti-tumor drug, is currently an integral part of treatment regime for OS along with methotrexate and doxorubicin. However, toxicity issues associated with combination module impede OS therapy. Also, despite the proven benefits of cisplatin, acquisition of resistance remains a concern with cisplatin-based therapy. This prompted us to investigate the molecular effects of cisplatin exposure and changes associated with acquired resistance in OS cells. Cisplatin shock was found to activate MAPK signaling and autophagy in OS cells. An activation of JNK and autophagy acted as pro-survival strategy, while ERK1/2 triggered apoptotic signals upon cisplatin stress. A crosstalk between JNK and autophagy was observed. Maximal sensitivity to cisplatin was obtained with simultaneous inhibition of both autophagy and JNK pathway. Cisplatin resistant cells were further developed by repetitive drug exposure followed by clonal selection. The resistant cells showed an altered signaling circuitry upon cisplatin exposure. Our results provide valuable cues to possible molecular alterations that can be considered for development of improved therapeutic strategy against osteosarcoma.

## Introduction

Osteosarcoma (OS) is the most prevalent primary malignant bone tumor, with an incidence peak predominant in adolescents and children [1]. It is an aggressive disease which when untreated shows rapid local and systemic progression leading to severe mortality. The 5-year survival rate of high grade OS or metastatic or recurrent disease is as low as 20%. In the past, despite, exceptional local control rates achieved through surgery, patients with even seemingly localized OS eventually developed metastasis and died [2]. The surgical failure and associated despondency necessitated the introduction and development of chemotherapeutic regimes for the treatment of OS. Currently, the gold standard treatment for OS includes pre-operative neo-adjuvant chemotherapy and also post-operative adjuvant chemotherapy [3]. However, in spite of an aggressive treatment regime, chemo-therapy is often rendered ineffective in OS due to acquired drug-resistance and associated disease relapse [4–6]. The OS cells are also reported to be inherently resistant to drugs. Endeavors to improve therapeutic efficacy by drug dose escalation or by alteration of chemotherapeutic drug combinations, have hardly improved the dismal survival outcome. Additionally, currently there is no standard chemo-therapy for OS that has relapsed post first-line multi-modal treatment [5–7]. This perpetually demands extensive study directed towards understanding the causes to drug-resistance to existing treatment modalities in OS which would facilitate identification of novel treatment targets to effectively subvert chemo-resistance and re-establish sensitivity in OS.

Cisplatin-based therapy either alone, or in combination with high-dose methotrexate and doxorubicin is widely used for OS treatment [8, 9]. However, multi-drug treatment is often associated with life-threatening toxicity, limiting its application [5, 6]. Hence, it is essential to identify novel molecules or pathways, crucial in cell survival, post cytotoxic drug exposure that can be targeted as a complement to conventional treatment. Such a strategy can reduce toxicity-associated effects of multi-modal treatments. In this study, we have explored the molecular bases behind cisplatin-associated resistance in OS; cisplatin (CDDP) is almost always used as neo-adjuvant chemotherapy in clinics for treatment regimes in high-grade OS. Despite the proven benefits of CDDP and being one of the most potent anti-tumor agents displaying clinical efficacy against a wide variety of tumors, a major stumbling block to CDDP success has been resistance to the drug restricting its application, OS is no exception [6, 10]. Hence, almost 30 years after the introduction of CDDP into clinical settings we are still in an effort to understand how to refine the therapeutic potential of CDDP. Independent studies need to be designed and conducted for each tumor type to understand and characterize the cause towards resistance to CDDP to improve the efficacy of this very potent drug. In this regard, existing literature provides insufficient information on the molecular mechanisms active post CDDP insult and resistance in human OS cells [11]. A paucity of appropriate model systems in OS, the rareness of the disease and inadequate access to patient material has probably been a hindrance to better biological understanding of this complex disease hampering development of effective therapy against OS.

This prompted us to develop a CDDP-resistant cell line model that mimics the condition OS patients experience during chemotherapy and evaluate the mechanisms underlying CDDP resistance in OS. Since, osteosarcoma is a rare disease; as a consequence, there are limited *in vitro* cell line models available for OS research compared to other cancers. However, the low prevalence of the disease renders these tumor-derived *in vitro* models highly precious for OS research [12]. We selected the parental HOS cell line for our study, since previous studies suggest that these cells are genetically more stable compared to highly metastatic OS cell derivates like, SAOS2 or LM7 [12]. The resistant cell line was characterized for its acquired resistance associated properties and molecular events leading to the same.

While dissecting the underlying causes to drug-resistance in OS it can be figured out that one of the major limitation to success of chemotherapy in OS is development of multi-drug resistance (e.g., over-expression of ABCB1) [13]. Though averting emergence of multi-drug-resistance has been a top priority in clinics and research investigations, yet, it has remained an elusive goal. Other major factors contributing to drug-resistance in OS has been cellular mechanisms like resistance to apoptosis and persistence of stem cell like characters in cancer cells [14]. Recently, O'Farrill and his group observed in a panel of OS cells that treatment efficacy to drugs like gemcitabine in OS is considerably decreased due to activation of protective autophagy [15]. However molecular pathways that govern the autophagy process in OS cells have not been thoroughly explored and a more thorough understanding of it may allow identification of possible biological markers that can be targeted for potential therapeutic benefits against OS.

Hence in our study, we have analyzed the molecular alterations in OS cells post CDDP shock and characterized underlying adaptations facilitating CDDP resistance in OS with special emphasis on role of autophagy and signaling governing it. The outcome of this study provides valuable insights into prospective therapies that can improve the sensitivity of OS to already existing treatments. To the best of our knowledge, we are the first group to report alterations in MAPK signaling and its regulatory control over cell death/survival strategies like, apoptosis and autophagy in OS cells post CDDP insult.

## Methods

### Chemical reagents

CDDP, chloroquine di phosphate (CQDP), and anti-β-actin antibody were purchased from Sigma. The MEK inhibitor, U0126 was purchased from Cell Signaling Technology. The JNK inhibitor, SP600125 and anti-rabbit secondary antibody was purchased from Santa Cruz Biotechnology. Primary antibodies were anti-rabbit and were procured from Cell Signaling Technology.

### Cell culture

Human cancer cell lines (HOS-CRL-1543 and HepG2) were obtained from NCCS, Pune, India, cultured at 37°C, 5% CO_2_, in minimal essential medium (MEM; HiMedia) supplemented with 10% fetal bovine serum (FBS; Invitrogen). Cells were typically grown to 60–70% confluence, rinsed in phosphate-buffered saline (PBS) and placed into fresh medium prior to treatments. To confirm the cell identity, DNA was isolated from the cells and was outsourced to Lifecode Technologies Pvt. Ltd., India for STR marker profile analysis.

### Creation of drug-resistant cell line

A human osteosarcoma cell line, resistant to CDDP (OS-R) was developed in our laboratory from HOS-CRL-1543. CDDP was dissolved in dimethyl sulfoxide (DMSO, SDFCL) for the treatment. According to reports by Hall *et al*, CDDP dissolved in DMSO can show a decreased activity when stored for a long period of time [16]. Hence, CDDP was freshly prepared each time before the experiments. Briefly, each 10-cm plates were seeded with 300,000 HOS cells, allowed to adhere overnight and then given a short pulse treatment of CDDP (1mg/ml) for 2h. The *in vitro* treatment dose for CDDP was determined based on effective dose used in clinical applications. Majority of the cells died post treatment, leaving a few, isolated, drug-tolerant, slow-growing cells (‘persisters’), with altered morphology on the plates. The surviving cells were given a PBS wash and allowed to revive in drug free media for a period of four weeks, with media being changed every 3 days during this period. Bright field images were also captured every 3 days with phase-contrast microscope to monitor the cells. After about a month, once the cells attained regular morphology, start growing and reach confluency, this cycle of short pulse treatment of CDDP followed by revival of cells in drug free media was repeated. The whole process was repeated three times to derive the OS-R cells. After each revival the cells were tested for their sensitivity to CDDP, by MTT assay, discussed below, to ascertain acquisition of drug-resistance. The OS-R cell derived from parental HOS-CRL-1543 were also periodically checked for their resistance property. The fold resistance was calculated by dividing the IC_50_ of resistant cell line / IC_50_ of parental cell line. OS-R cells were maintained and cultured under IC_50_ drug pressure. The repetitive pulsed treatment strategy, for establishment of resistant cells, where cells are given a short treatment pulse and allowed to recover in drug-free media, was chosen over escalating dose strategy, to develop a clinically relevant resistant model.

### Cell viability assays

*In vitro* cytotoxicity was performed by 3-(4,5-dimethylthiazol-2-yl)-2,5-di-phenyltetrazolium bromide (MTT) assay, as described previously by Chowdhury et al [17]. Briefly, cells were cultured in 96-well plates overnight. The following day, cells were treated with drugs or compounds for desired period of time. Thereafter, MTT was added to each treated and control well and incubated for 4h. Formazan crystals were solubilized in DMSO and readings were obtained at 495nm with a differential filter of 630nm using a micro-plate reader (Start-fax 2100). Percentage of viable cells was calculated using formula: viability (%) = (mean absorbance value of drug-treated cells) / (mean absorbance value of control) *100. A concentration of 0.2% DMSO was found to be non-toxic and was used for dissolving CDDP, and used as control in cytotoxicity experiments. Wherever mentioned, cell viability was also analyzed by trypan blue dye exclusion assay. Viable cells do not take up the dye, but dead cells are permeable and take up the dye. Following specific treatments, cells were stained with trypan blue (0.4%) and proportion of dead and viable cells were counted in a cell counter (TC20^™^ Automated Cell Counter, Bio-Rad).

### Single cell colony formation assay

In order to determine the colony formation capacity of HOS and OS-R cells, they were serially diluted in 96-well plate until groups of scattered single cells were observed. The plate was then monitored for the number of colonies formed for around 10 days. The number of colonies were manually counted.

### Collection of conditioned media (CM)

For collection of CM, HOS and OS-R cells were plated in 25cm^2^ flasks, washed twice with PBS, and incubated for 48h with fresh media. The supernatant was harvested, centrifuged at 3,000rpm for 5min to remove suspended cells and then stored at −80°C for later use. In order to confirm that any change in survival post incubation with CM is not due to degradation of CDDP, we followed procedure described by Schuldes et al 1997 for CM related experiments [18].

### RNA isolation and real-time PCR (RT-PCR)

Total RNA was isolated using TRIzol reagent (Invitrogen); complementary DNA (cDNA) was synthesized using GeneSure First Strand cDNA Synthesis kit (Genetix) with oligodT following manufacturer's instructions. cDNA templates were amplified for ABCB1 gene in CFX Connect RT-PCR System (Bio-Rad) and detected using SYBR Green (Bio-Rad). As a control, GAPDH was amplified. The primer sequence for ABCB1 was forward -5’- GGGATG GTCAGTGTTGATGGA -3’ and reverse 5’-GCTATCGTGGTGGCAAACAATA-3’ and for GAPDH was forward- 5’-TGATGACATCAAGAAGGTGGTGAAG-3’ and reverse- 5’-TCC TTGGAGGCCATGTGGGCCAT-3’. The relative RNA expression was calculated using the Livak method [19].

### Immunoblotting

Immunoblotting was performed following protocols as described previously [20]. Cells were lysed in modified RIPA buffer (Sigma-Aldrich) and protein content was measured using Bradford reagent (Thermo Scientific). The cellular protein lysates were run in denaturing polyacrylamide gels and thereafter transferred to PVDF membrane (Millipore) for blocking with 5% skimmed milk (HiMedia). The blots were then probed or re-probed with specific primary antibodies and detected using enhanced chemi-luminescence (ECL) detection system following the manufacturer's protocol. The primary antibodies used were as follows: anti-p38, anti-JNK, anti-ERK1/2, anti-PARP-1, anti-ATG and anti-LC3B-II. The secondary antibodies were horseradish peroxide-conjugated goat anti-rabbit IgG. Expression was quantitated using ImageJ software.

### Staining with monodansylcadaverine (MDC)

The drug monodansylcadaverine (Sigma), a specific autophagolysosomal marker was used to analyze autophagy. For visualization of autophagic vacuoles by microscopy, cells were plated on cover slips overnight. The following day, the cells were treated with CDDP. Post treatment, cells were incubated for 10min with 0.05mM MDC in PBS at 37°C. After incubation, the cover slips containing the cells were washed with PBS and mounted with anti-fade mountant (containing DAPI). Intracellular MDC in the form of punctate dots were analyzed by fluorescence microscopy. A simultaneous fluorimetric measurement, after labeling the cells with MDC was also carried out. Cells labeled with MDC for 10 min, were washed with PBS and collected in 10mM Tris-HCl (pH 8) containing 0.1% TritonX-100. Intracellular MDC was measured by fluorescence photometry (excitation 380nm and emission 525nm) in a microplate reader (Fluoroskan Ascent). An increase in MDC fluorescence upon treatment was expressed as fold change with respect to control.

### Flow cytometric analysis of apoptotic cells

For determination of apoptosis, HOS cells were seeded in 6cm dishes at a density of 1 × 10^6^ cells. The cells were then exposed to various treatments. Thereafter, the cells were harvested, washed with PBS and re-suspended in 500μL of 1X binding buffer (BD BioSciences). Thereafter, 4μl of AnnexinV-FITC and 10 μl of Propidium Iodide (PI) were added to the cells in binding buffer, followed by incubation in dark for 20min. The samples were then acquired using flow cytometer (Cytoflex, Beckmann Coulter) and analysis of acquired data was performed using CytExpert software. To detect both early and late apoptotic cells, the percentage of cells in lower and upper right (LR and UR) quadrant representative of only AnnexinV and both AnnexinV/PI positive cells respectively, were counted. A fold increase in apoptotic cells is represented through bar diagram.

### Caspase activity assay

For measurement of caspase activity, 1 × 10^4^ cells/well were seeded and exposed to different combination of treatments for 24h. For each treatment condition, cells were also exposed to caspase inhibitor Z-VAD-FMK (carbobenzoxy-valyl-alanyl-aspartyl-[O-methyl]—fluoromethylketone, 50μM, Promega), a cell-permeable pan caspase inhibitor that irreversibly binds to the catalytic site of caspase proteases and inhibit induction of apoptosis. The caspase inhibitor was added 1h prior to treatments. The activity of caspase-3 was then measured using caspase-3 colorimetric protease assay kit (Invitrogen) following the manufacturer’s protocol. Briefly, the cell lysate was collected in RIPA buffer and the concentration of protein was determined using Bradford assay. Equal amount (60 μg) of protein mixed with reaction buffer was added to microtiter plates following incubation with caspase-3 substrate (acetyl-Asp-Glu-Val-Asp p-nitroanilide, Ac-DEVD-pNA) for 1 h. The absorbance was read at 405 nm using an micro-plate reader (Start-fax 2100, Awareness Tech. Ltd). The colorimetric assay is based on the hydrolysis of caspase-3 substrate by caspase-3 enzyme, resulting in the release of the p-nitroaniline (pNA) moiety. The concentration of the pNA released from the substrate was calculated from the absorbance values at 405 nm.

### Transfection of wild type p53

A pCMV-Neo-Bam mammalian expression vector containing a 1.8kb fragment encoding wild-type p53 (from nucleotide -130 to 1671 relative to the start codon) driven by CMV promoter was transiently transfected into the HOS cells with Lipofectamine 2000 following the manufacturer's instructions (pCMV-Neo-Bam p53 vector was a kind gift from Bert Vogelstein, Addgene plasmid # 16434). After 24h of transfection, cells were lysed in RIPA buffer and analyzed for specific proteins through immunoblot following procedure described above.

### Statistical analysis

Tukey tests was used as a follow up to one way or two way ANOVA to compare every mean to a control mean and every mean with every other mean using Graph Pad Prism software version 5.0. The Bonferroni method was used to analyze multiple comparisons using Graph Pad Prism software version 5.0. The tests compute a confidence interval for the difference between the two means. The (*) in figures denotes a significant change with reference to compared sample at 95% confidence level.

## Results

### HOS cells surviving CDDP shock show altered drug sensitivity

**Fig 1:**
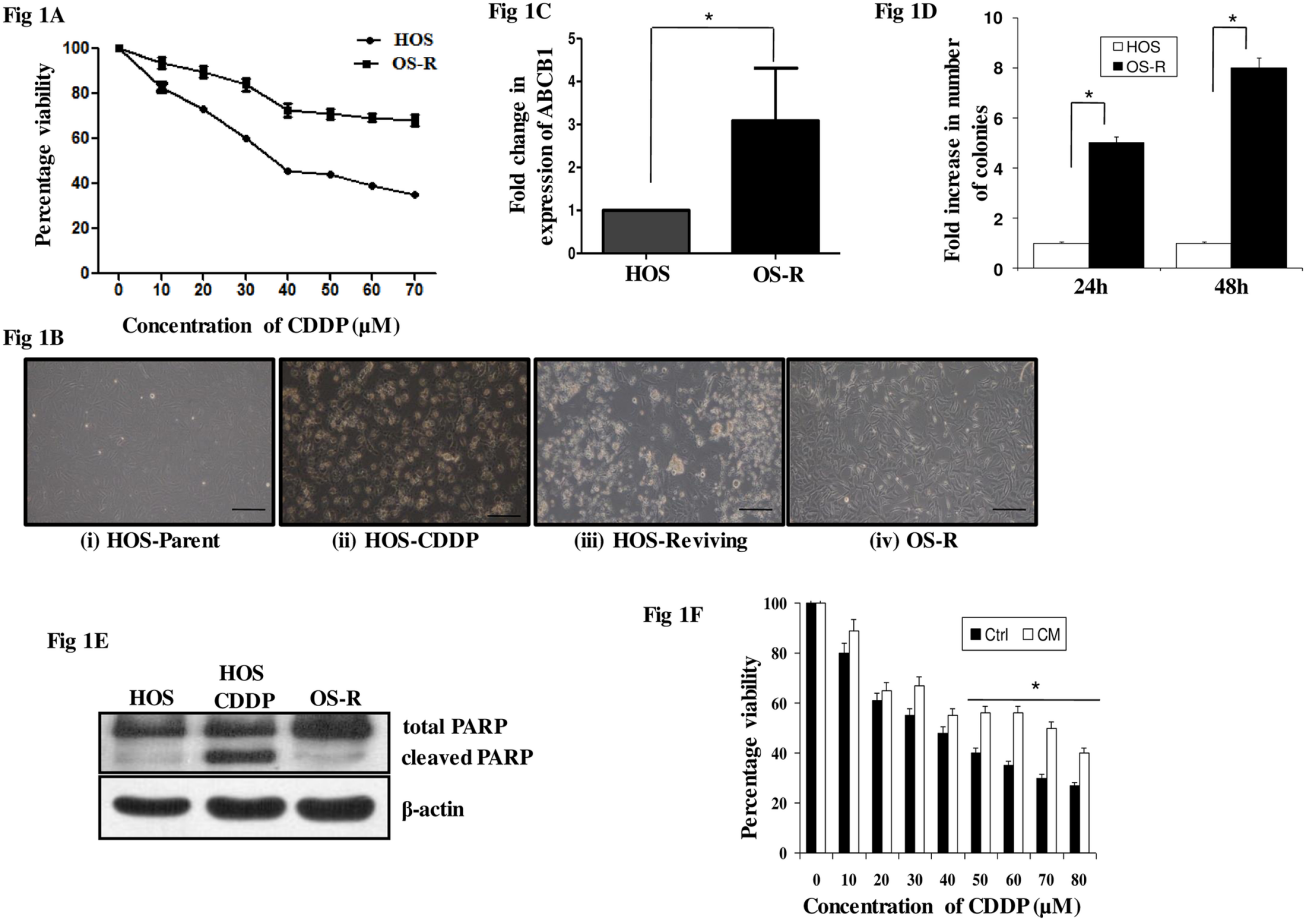
Development and characterization of HOS-resistant model. (A) Cells were seeded at a density of 4000 per well in triplicates. MTT assay was performed following CDDP exposure for 24h. Percentage viability of OS-R cells compared to parental cells is represented in the figure. (B) Phase contrast images of—i. HOS parental untreated cells; ii. HOS-CDDP-treated cells, images were captured after 30min of 1mg/ml CDDP shock; iii. HOS cells reviving post shock when maintained in drug free media for around 3–4 weeks; iv. Resistant cells (OS-R) maintained in IC_50_ dose of CDDP. The scale bar represents 30μm. (C) cDNA was synthesized from DNase-treated RNA isolated from HOS and OS-R cells. RT-PCR was performed with ABCB1 specific primers. Fold change in expression level of ABCB1 gene is represented in the figure with untreated control taken as “1”. (D) Single cell colony formation assay was performed in HOS and OS-R cells. Cells were counted and serially diluted until distinct single cells were observed. The cells were then monitored for their colony forming capacity. Fold increase in number of colonies formed after 24 and 48h in OS-R cells compared to HOS is represented. (E) Levels of cleaved PARP-1 was monitored by immunoblot after IC_50_ dose of CDDP exposure for 24h in HOS cells and in OS-R cells maintained in IC_50_ dose of CDDP. (F) The HOS cells were maintained in conditioned media (CM) for 48h and survivability upon exposure to various concentrations of CDDP was measured by MTT assay. Here, control (Ctrl) represents regular media in which HOS cells were cultured. The symbol (*) signifies a significant difference in CM with respect to control.

Human osteosarcoma cell, HOS was exposed to increasing doses of CDDP to evaluate drug sensitivity. The inhibitory concentration of CDDP at which 50% of HOS cells died (IC_50_) was found to be 35μM through MTT assay (Fig 1A). Significantly increased cell viability (80%) was obtained at IC_50_ concentration for the cells derived by consecutive high dose exposure to CDDP (Fig 1A). The fold resistance calculated following methods described in materials and methods was found to be 2.2. Currently, there are multiple methodologies used by researchers to create drug resistant cells. For example, the "incremental method" involves selecting an initial low dose and exposing the cells to that dose for a given period of time. After one or more treatments at that low dose, the drug concentration is increased and the cycle is repeated. This is in contrast to a lesser used method called the “pulse method" [21, 22]. The pulse method closely resembles the weekly intra-venous pulse dosing regimen prescribed for cancer patients. In this method, the cells are periodically exposed to an unchanged dose without increasing its initial concentration. When the two different treatment protocols are compared with the end goal of determining a better clinical model, it is observed that the pulse method better simulate resistance observed in patients undergoing chemotherapy and may hence serve as a superior model to investigate cellular drug-resistance [21–23]. To determine the level of drug resistance to be achieved we also referred to existing literature describing cell lines that were established from cancer patients before and after chemotherapy [23]. In majority of these cell lines developed from patients post-chemotherapy, a two- to five-fold increase in resistance to the anti-cancer agents was observed [23]. Though, high-level models, often achieved by escalating dose strategy (incremental method) are kind of more stably resistant and easier to maintain in culture for prolonged period, the levels of resistance are often higher and as such molecular changes associated with resistance is less relevant to clinical settings [21–23]. Hence, to mimic clinical conditions a twofold resistance was achieved through pulse treatment strategy and selected for further studies. Morphological alterations associated with acquisition of resistance in HOS cells, is represented in Fig 1B. An increased expression of multi-drug resistant protein is one mechanism by which cells acquire resistance to chemo-therapeutics. Hence, the relative expression of ATP-dependent drug efflux pump ABCB1 was verified through quantitative RT-PCR. The expression level was found to be 3.085 fold higher in drug-resistant cells (OS-R) (Fig 1C). There are previous reports of ABCB1 over-expression contributing to resistance in OS [13]. Cross-resistance to other drugs often parallels insensitivity to CDDP indicating a common mechanism employed by cells to gain resistance against anti-cancer drugs. In our study the OS-R cells showed cross-resistance to the thymidine analogue, 5-flurouracil (5-FU). The IC50 for 5-FU in HOS cells was found to be 40μM; an approximately 17% more cell viability was observed at the 5-FU-IC50 dose for OS-R cells. To compare the proliferation efficiency of HOS and OS-R cells single cell clonogenic assay was performed. The number of colonies formed after 24h and 48h was 5 and 8 fold higher in OS-R cells respectively compared to parental cells indicating an increased proliferative potential of the cells (Fig 1D). Furthermore, CDDP treatment (IC_50_) induced poly-ADP-ribose-polymerase-1 (PARP-1) cleavage in HOS cells, an indicator of DNA damage and apoptotic cell death, but not in OS-R (Fig 1E). The OS-R cells used were maintained at IC_50_ concentration of CDDP. Also, when the OS-R cells were trypsinized, re-plated and treated with IC_50_ dose of CDDP they showed no change in cleaved PARP-1 expression levels compared to untreated control (not shown). Phase contrast images depicting insignificant effect of CDDP on cell viability of OS-R cells is shown in S1A Fig. Furthermore, ELISA based caspase-3 activity also showed that CDDP treatment in OS-R cells has no effect on the enzymatic activity levels (S1B Fig). Interestingly, media from OS-R culture (CM) induced CDDP-resistance in parental cells, when cultured in CM for 48h (Fig 1F). The OS-R cells probably secrete components in the media, which has the potential to impart drug-resistance to parental cells. Currently we are in the process of exploring proteins and exosomal components differentially secreted in the conditioned media by the OS-R cells compared to parental control.

### Inhibition of autophagy significantly sensitized the parental HOS cells to CDDP

**Fig 2:**
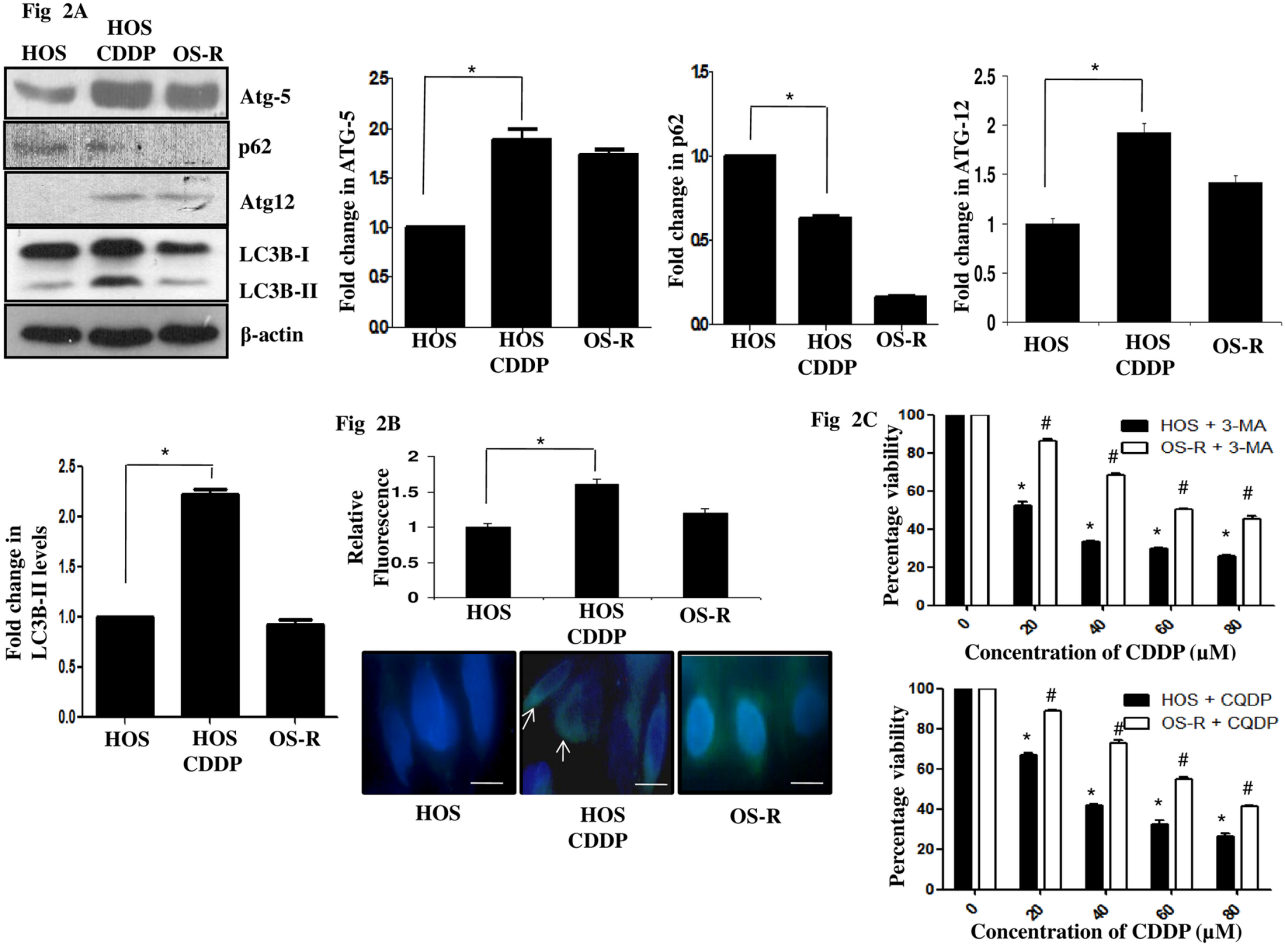
Effect of CDDP on autophagy in HOS and OS-R cells. (A) Immunoblot analysis was performed to analyze expression of specific autophagic markers representing late stages of autophagy following CDDP exposure (IC_50_) for 24h in HOS cells. Densitometric scanning of each blot was performed with ImageJ software to quantitate differences in expression. Expression level in untreated control HOS cells was set as arbitrary unit “1” in this and in subsequent figures. β-Actin served as a loading control. (B) MDC fluorescence staining was performed in HOS cells with and without treatment with CDDP (IC_50_) for 24h and in OS-R cells. Green dots indicative of autophagosomes were monitored by fluorescence microscopy. A representative image is presented. Fluorimetric measurement of MDC fluorescence is also presented in the form bar diagram, where control was considered as arbitrary unit "1". (C) HOS and OS-R cells cultured in 96-well plates were pre-treated for 24h with autophagy inhibitors, 3-MA (8μM) and CQDP (10μM) and then exposed to various concentrations of CDDP. MTT assay was performed for each sample in triplicate. The graph represents percentage viability of cells. The symbol (*) denotes a significant difference with respect to untreated HOS cells and the symbol (^#^) denotes a significant difference in OS-R with respect to HOS cells.

**Fig 3:**
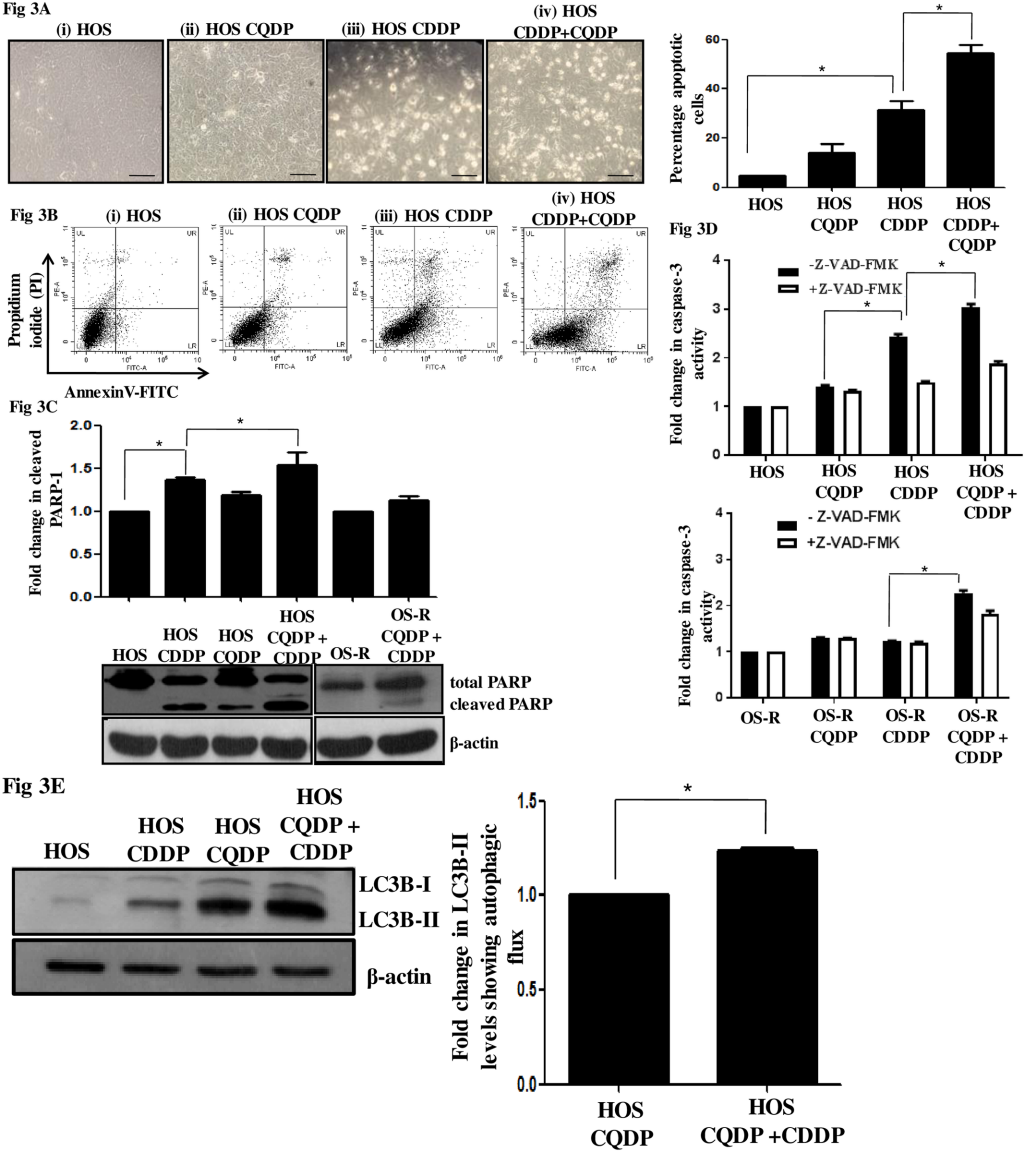
Effect of autophagy inhibition in HOS and OS-R cells. (A) Phase contrast images showing morphology of HOS untreated (i), CDDP (IC_50_) treated (ii), only CQDP (10μM)-treated (iii) and cells treated for 24h with both CQDP and CDDP (iv). The scale bar represents 30μm. (B) Flow cytometric analysis of apoptosis with AnnexinV/PI upon autophagy inhibition. HOS cells were either untreated (i) or treated with CDDP (IC_50_) (ii), only CQDP (10μM) (iii) or both CQDP and CDDP (iv) for 24h. CQDP was added 24h prior to CDDP treatment. Cells present in the lower right (LR) and upper right (UR) quadrant represent early and late apoptotic cells respectively. A fold increase in total number of apoptotic cells upon different treatment is represented through bar diagram. (C) Immunoblot showing cleaved PARP-1 expression levels in HOS and OS-R cells after 24h of CDDP exposure in presence or absence of CQDP (10μM). Densitometric scanning was performed with ImageJ software. β-Actin served as a loading control. (D) Caspase-3 assay was performed to determine apoptosis induction in cells after various treatments. A prior treatment of CQDP (10μM, 24h) was given to the cells before CDDP (IC_50_) exposure. To inhibit caspase activity Z-VAD-FMK was used at 50μM concentration and was added 1h prior to administration of various treatments. Activity in control HOS and OS-R cells was taken as "1". (E) Autophagic flux representative of difference in protein level of LC3B-II between CQDP treated HOS cells and CQDP plus CDDP (IC_50_) treated cells was checked by immunoblot. The same is represented with bar diagram. A prior treatment of CQDP (10μM, 24h) was given to cells before CDDP exposure.

Autophagy is an evolutionary conserved intracellular process that degrades and recycles cellular nutrients. It is often activated upon stress stimuli [24, 25]. Existing evidences suggest a role of autophagy in hindering drug sensitivity, however, it is controversially discussed [26, 27]. In OS cells too, cyto-protective autophagy was found to be active in a series of cell lines upon gemcitabine treatment, however gemcitabine is not part of current treatment regime for OS. Hence, detailed study on drugs like CDDP or methotrexate, which are often used for sensitization of OS cells, should be carried out for evaluating their potential of triggering autophagic pro-survival strategy in OS cells. This may provide critical insights into survival mechanisms that might be induced post drug exposure in a clinical setting. Recently, Zhang et al observed autophagy activation in MG63 human osteosarcoma cell line under drug stress, however, detailed study on the same was not explored [28]. We therefore initially analyzed for activation of autophagy upon CDDP exposure in the osteosarcoma cells. An increase in microtubule-associated protein light chain 3B-II (LC3B-II), indicative of autophagic activity was observed in HOS cells after CDDP treatment (Fig 2A). Immunoblot assessment of the increase in LC3B-II form is currently considered as a simple and quick procedure to quantify cellular autophagy (2) and its use is advised in published guidelines for interpretation and monitoring of autophagy (8). However, OS-R cells failed to show a similar effect (Fig 2A and S1C Fig). Also, there was an increase in protein levels of ATG-5 and ATG-12, which forms a complex with ATG-16 to enable autophagosome formation and lipidation of LC3. Simultaneously, a decrease in p62, the turnover of which serves as a useful marker for induction of autophagy was obtained following exposure of CDDP (Fig 2A). Interestingly, basal level of autophagy was also found to be high in OS-R cells compared to untreated HOS cells. However, no alteration in autophagic protein (ATG-5 and LC3B-II) levels was observed in OS-R cells when treated with CDDP (S1C Fig). The drug monodansylcadaverine (MDC), a specific autophagolysosomal marker was also further used to analyze autophagy induction upon CDDP addition. A significant increase in MDC fluorescence evident as green dots in Fig 2B, was observed in HOS cells upon CDDP treatment (Fig 2B). There exists confusion on the precise role of autophagy (pro-death or pro-survival) upon drug insult. Autophagy was hence inhibited with selective autophagy inhibitor, like, chloroquine di phosphate (CQDP), which is known to hinder the fusion of autophagosome with lysosome. Interestingly, pre-treatment with CQDP increased CDDP sensitivity more significantly in HOS cells than OS-R cells (Fig 2C). Similar results were obtained with 3-methyl adenine (3-MA), which is known to inhibit early autophagic processes (Fig 2C). However, since 3-MA is an upstream autophagy inhibitor and previous reports suggest that 3-MA can inhibit both class I and III phosphoinositide 3-kinase (PI3K) activity thus having a role in both activating or inhibiting autophagy, we selected CQDP for subsequent experiments [29]. Morphological alterations observed following CQDP and CDDP exposure in HOS cells were also indicative of increased cell death (Fig 3A). To check whether autophagy inhibition increases apoptosis, we analyzed presence of apoptotic cells through flow cytometry using AnnexinV/PI staining. An increase in percentage of apoptotic cells was observed upon CDDP-treatment; however, upon autophagy inhibition a further significant increase in percentage apoptotic cells was observed in HOS cells compared to only CDDP treatment (Fig 3B). To further confirm apoptosis, PARP-1 cleavage was analyzed through immunoblot and caspase-3 activation by ELISA based method. An inhibition of autophagy by CQDP followed by CDDP treatment significantly induced the both cleaved PARP and caspase activity, compared to only CDDP treatment in HOS cells (Fig 3C and 3D). Caspase activity was inhibited with caspase inhibitor, Z-VAD-FMK to confirm specific activation of caspases. Interestingly, the changes in PARP-1 or caspase-3 activity in CDDP and CQDP treated HOS cells were significantly higher as compared to OS-R cells treated with same compounds (Fig 3C and 3D). This signifies that, post acquisition of drug resistance, inhibition of autophagy upon drug stress has a less drastic effect on cell sensitivity. Since, the accumulation of autophagosomes is not always indicative of autophagy induction and may just represent either the increased generation of autophagosomes and/or a block in autophagosomal maturation, we hence checked for the difference in the amount of LC3B-II in the presence or absence of CQDP in HOS cells [30]. The difference in the amount of LC3B-II between samples in the presence and absence of lysosomal inhibitors (e.g., CQDP) represent the amount of LC3 that is delivered to lysosomes for degradation (i.e., autophagic flux). An increase in LC3B-II level, indicative of autophagic flux, was observed in cells treated with CQDP plus CDDP compared to only CQDP treated cells (Fig 3E). The above experiments are suggestive of the fact that autophagy is induced in both HOS and OS-R cells upon CDDP exposure, but the HOS cells are comparatively more sensitive to autophagy inhibition than OS-R.

### Activated ERK1/2 induces apoptosis upon drug stress in HOS cells

**Fig 4:**
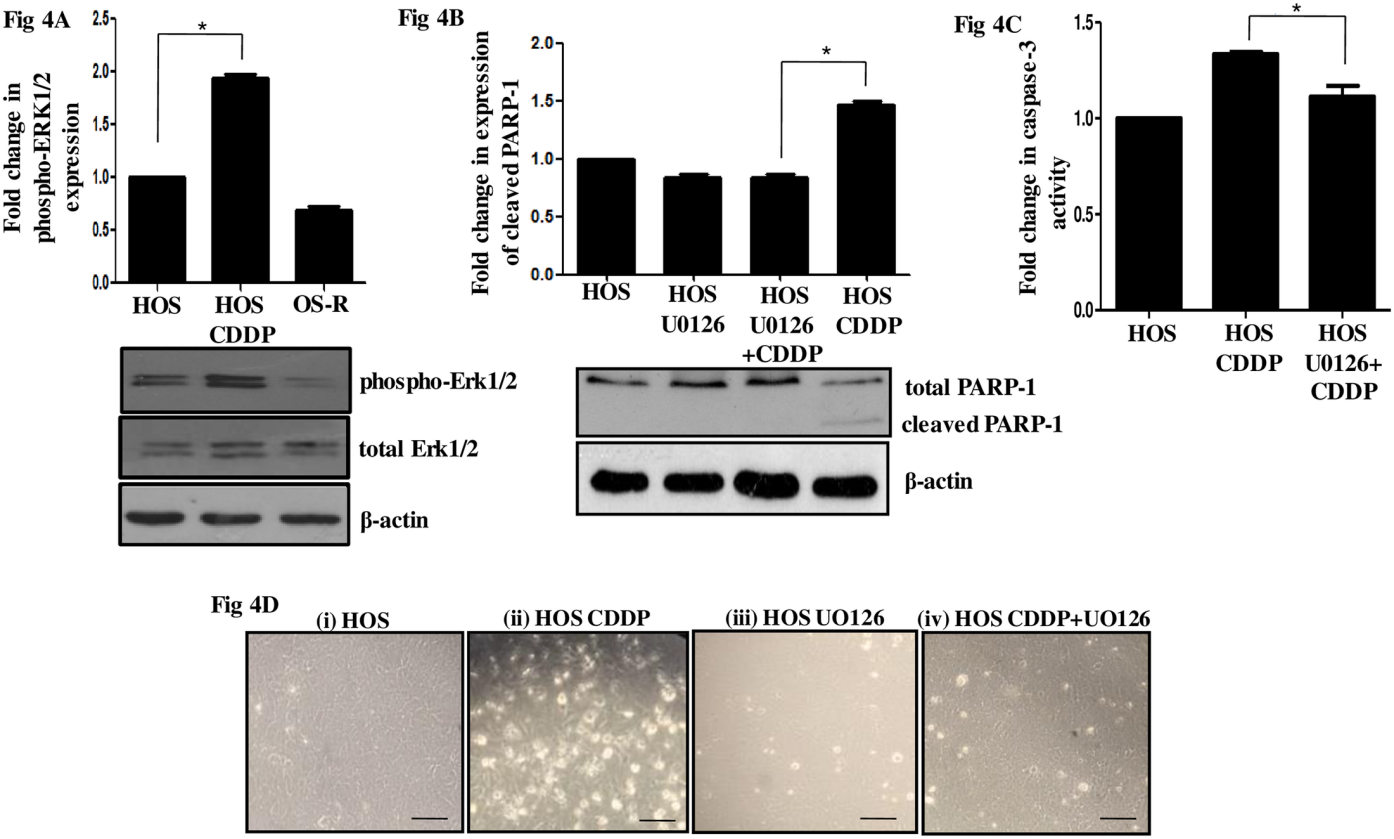
Role of activated ERK1/2 signaling upon CDDP stress. (A) Levels of phosphorylated-ERK1/2 were analyzed by immunoblot after CDDP exposure (IC_50_) for 24h in HOS cells. OS-R cells were maintained at similar CDDP concentration. Bands were densitometrically scanned with ImageJ and normalized to total ERK1/2. β-Actin served as a loading control. (B) ERK1/2 signaling was inhibited in HOS cells with U0126 (10μM; 2h) and CDDP (IC_50_) treatment was made thereafter for 24h. Cleaved PARP-1 levels were then analyzed by immunoblot. β-Actin served as a loading control. (C) A fold change in caspase-3 activity was analyzed colorimetrically in HOS cells. ERK1/2 signaling was inhibited with U0126 (10μM; 2h) followed by CDDP (IC_50_) treatment for 24h. Activity in control HOS cells was considered as "1". (D) Representative phase contrast images for HOS-untreated (i), CDDP (IC_50_)-treated (24h) (ii), only U0126-treated (iii), and cells with prior ERK1/2 inhibition followed by CDDP (IC_50_) treatment (24h) (iv). The scale bar represents 30μm.

An alteration in MAPK signaling pathway is a prominent feature of many cancers; it is well-known to link extracellular inputs to cellular processes that can control growth, proliferation, migration and apoptosis depending on the cell type and microenvironment [31]. In this study, we observed an alteration in MAPK signaling upon CDDP stress in HOS cells. An increased phosphorylation at Thr202/Tyr204 of p44/42 MAPK (ERK1/2) indicative of its activation was observed in drug-treated cells (Fig 4A). Interestingly, the OS-R cells did not show a similar activation when maintained or exposed at the same drug dose (Fig 4A and S1D Fig). To understand the role of the activated ERK1/2, we pharmacologically inhibited it with U0126, a selective inhibitor of the upstream MAP kinase kinases. A prior inhibition of the ERK1/2 signaling followed by CDDP treatment resulted in decreased PARP-1 cleavage and caspase-3 activity compared to only CDDP-treated HOS cells (Fig 4B and 4C). The morphological alterations as observed upon ERK1/2 inhibition under CDDP stress is shown in Fig 4D. A distinct decrease in rounded up apoptotic cells were observed in CDDP-treated-ERK1/2-inhibited cells compared to only CDDP-exposed cells. This clearly indicates that ERK1/2 plays a pro-apoptotic role in these cells under CDDP stress and ERK1/2 inhibition inhibits apoptosis.

### Activation of JNK signaling opposes pro-apoptotic effect and increases autophagic flux

**Fig 5:**
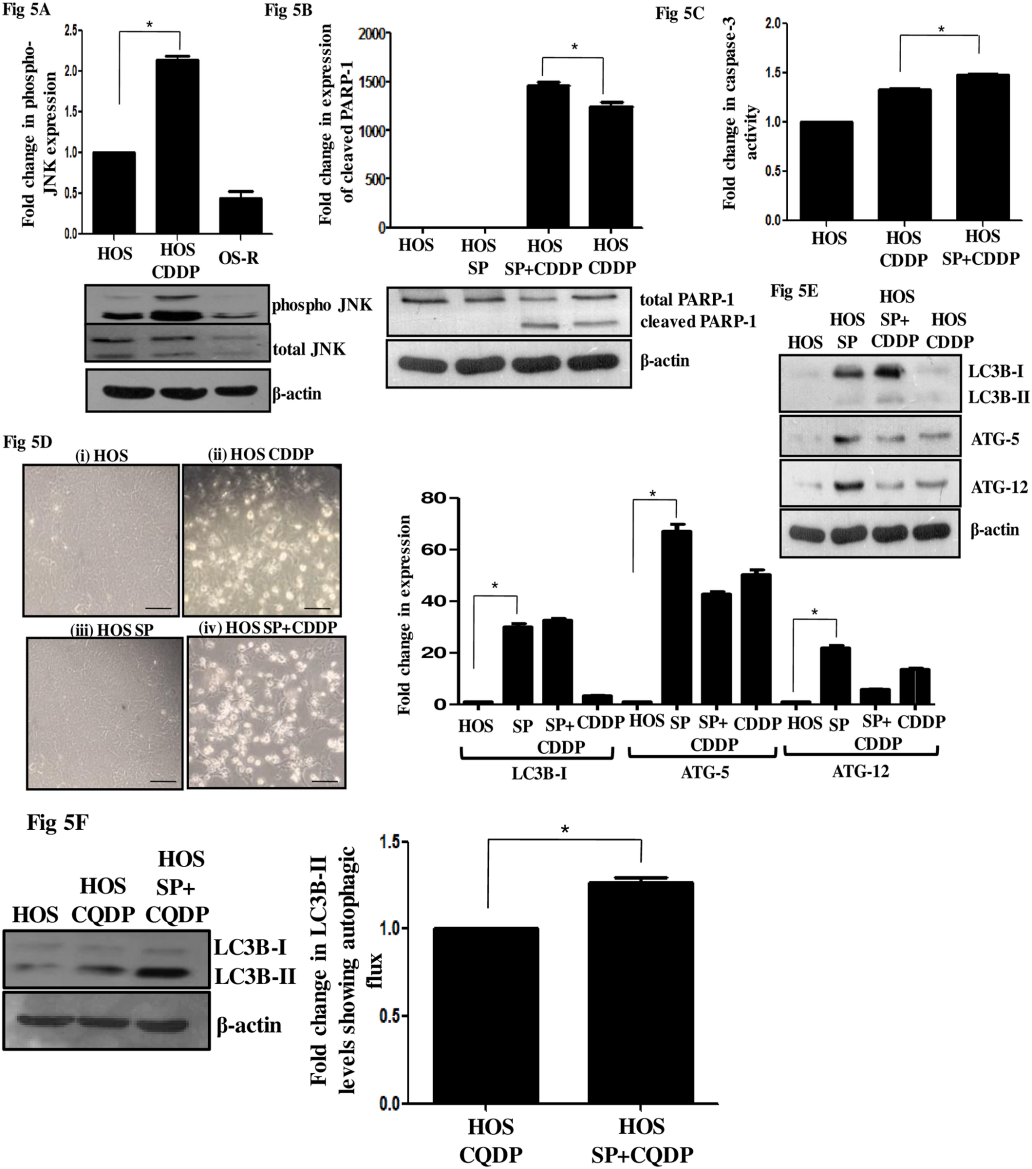
Role of activated JNK signaling upon CDDP stress. (A) Levels of phosphorylated-JNK were analyzed by immunoblot in untreated HOS cells, in CDDP exposed (at IC_50_ dose) HOS cells for 24h and in OS-R cells. Densitometric scanning was performed with ImageJ software and normalized to total protein. β-Actin served as a loading control. (B) JNK signaling was inhibited in HOS cells with SP600125 (SP, 25μM). SP was added 2h prior to CDDP treatment (IC_50_ for 24h). Cleaved PARP-1 levels were then analyzed by immunoblot in untreated HOS, only SP-treated cells, only CDDP-treated cells, and in HOS cells treated with SP plus CDDP. β-Actin served as a loading control. Densitometric scanning was performed with ImageJ software. (C) A fold change in caspase-3 activity was analyzed colorimetrically in HOS cells. JNK signaling was inhibited with SP (25μM; added 2h prior to CDDP) followed by CDDP (IC_50_) treatment for 24h. Activity in control HOS cells was considered as "1". (D) Representative phase contrast images for HOS-untreated (i), CDDP (IC_50_)-treated (24h) (ii), only SP-treated (iii), and cells with prior JNK inhibition followed by CDDP (IC_50_) treatment (24h) (iv). The scale bar represents 30μm. (E) Autophagy was inhibited in HOS cells with prior treatment of CQDP (10μM, 24h) followed by CDDP (IC_50_) exposure. Protein expression of autophagic markers- LC3B-II, ATG-5 and ATG-12 were analyzed by immunoblot. Densitometric scanning was performed with ImageJ and represented. β-Actin served as a loading control. (F) To confirm an increase in autophagic flux upon JNK inhibition HOS cells were treated with SP (25μM; 2h) followed by CQDP (10μM) for 24h. Immunoblot analysis was performed for anti-LC3B-II. Difference in protein level of LC3B-II is represented with a bar diagram. β-Actin served as a loading control.

In parallel to ERK1/2 activation, activating phosphorylation (Thr183/Tyr185) of c-Jun NH2-terminal kinase (JNK) was observed following CDDP exposure (Fig 5A). However, the OS-R cells did not show a similar activation when maintained or exposed at the same drug dose (Fig 5A and S1D Fig). JNK pathway is often implicated in defense against oxidative and xenobiotic insults. It is also well known to activate stress-induced apoptosis [32]. However, JNK knockout mice are embryonically lethal due to an increased apoptosis of liver, indicative of their critical role in survival [32]. Hence, we were inquisitive to understand the role JNK activation upon CDDP stress in HOS cells. A pharmacological inhibition of JNK with SP600125 (SP) increased the expression of cleaved-PARP-1 as analyzed through immunoblot (Fig 5B). Also, an increased level of caspase-3 activity was observed in JNK-inhibited CDDP-treated cells compared to only CDDP-treated cells, as measured through ELISA-based assay (Fig 5C). Morphological alterations observed upon JNK inhibition further confirm the pro-survival role of JNK under CDDP stress in HOS cells (Fig 5D). Distinctly more cell death was observed in SP plus CDDP-treated cells. Thus, ERK1/2 and JNK play an antagonistic role under drug pressure in HOS cells. The resistant cells however, failed to show any significant activation of the MAPKs when maintained at similar concentration of CDDP.

JNK pathway has been previously linked to regulation of autophagy at multiple levels. JNK can inhibit Bcl_2_-Beclin-1 interaction leading to release of Beclin-1, the autophagic effector. JNK has also been implicated in autophagy-induced cell death through p62 accumulation [33]. Hence, we were interested in understanding whether JNK activation and autophagy induction, observed under CDDP stress, has any link. Interestingly, protein levels of LC3B-I and LC3B-II, indicative of an autophagic flux, increased upon JNK inhibition by SP (Fig 5E). Klionsky *et al* in 2016 refers to the fact that LC3I accumulation post an autophagy inhibitor can also be a true indicator for actual change in autophagic flux and not just change in levels of LC3B-II [30]. Similarly, a stark increase in ATG-5 and ATG-12 was also observed with SP exposure. To confirm whether an increase in LC3B is indicative of enhanced autophagic flux, we inhibited autophagolysosomal maturation by CQDP and observed an increase in LC3B-II levels in SP plus CQDP treated samples compared to only CQDP (Fig 5F). These results clearly show that JNK inhibition can induce autophagy under CDDP stress in these cells.

### Autophagy inhibition caused a time-dependent increase in phospho-JNK levels

**Fig 6:**
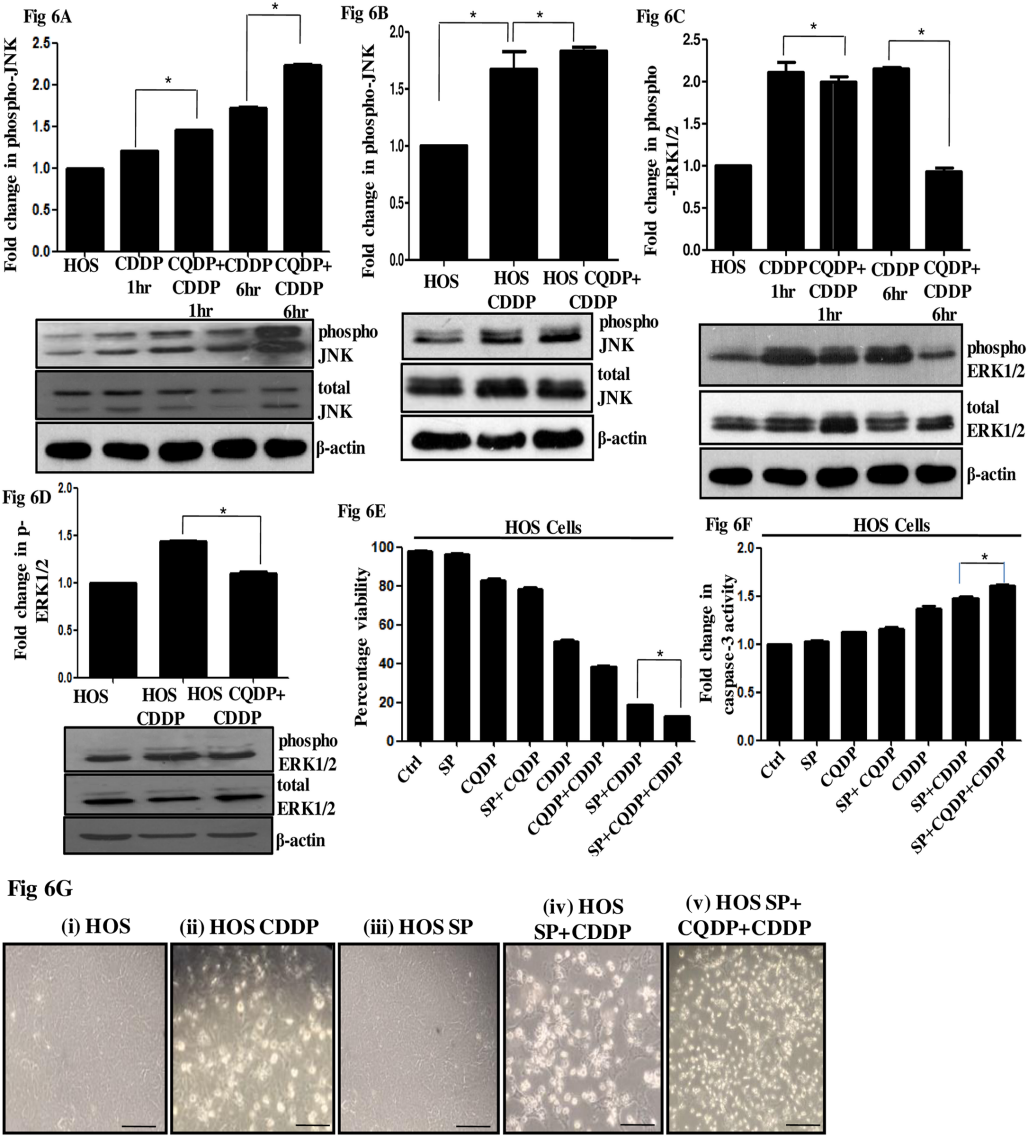
Effect of autophagy inhibition on MAPK signaling. (A) Levels of phosphorylated-JNK were analyzed by immunoblot after CDDP exposure (IC_50_) for 1h (A), 6h (A) and 24h (B) in HOS cells. A prior treatment of CQDP (10μM, 24h) was given wherever mentioned before CDDP exposure. Densitometric scanning was performed with ImageJ. β-Actin served as a loading control. (C and D) Levels of phosphorylated-ERK1/2 were analyzed by immunoblot after CDDP exposure (IC_50_) for 1h (C), 6h (C) and 24h (D) in HOS cells. A prior treatment of CQDP (10μM, 24h) was given wherever mentioned before CDDP exposure. Densitometric scanning was performed with ImageJ software. β-Actin served as a loading control. (E) Cell viability was measured 24h after treatment by trypan blue assay. Cells were either untreated or treated with the following:- SP (25μM); CQDP (10μM); SP (25μM) plus CQDP (10μM); CDDP (IC_50_); CQDP (10μM) plus CDDP (IC_50_); SP (25μM) plus CDDP (IC_50_); or SP (25μM) plus CQDP (10μM) plus CDDP (IC_50_). (F) A fold change in caspase-3 activity was analyzed colorimetrically 24h after treatment in HOS cells. Cells were either untreated or treated with the following:- SP (25μM); CQDP (10μM); SP (25μM) plus CQDP (10μM); CDDP (IC_50_); SP (25μM) plus CDDP (IC_50_); or SP (25μM) plus CQDP (10μM) plus CDDP (IC_50_). Activity in control HOS cells was considered as "1". (G) Representative phase contrast images of HOS cells treated with CDDP (IC_50_) for 24h or along with combinations of inhibitors, SP600125 (25μM) or CQDP (10μM) or both (i-v). The scale bar represents 30μm.

To further investigate the cross talk between JNK signaling and autophagy, we inhibited autophagy with CQDP and studied JNK and ERK1/2 activation over time under CDDP stress. Interestingly, increased JNK phosphorylation was observed upon autophagy inhibition. The activation was significantly high at 6h compared to 1h and 24h (Fig 6A and 6B). This signifies that the effect of the inhibitor was time dependent and more significant at early exposure hours. In contrary, the level of phospho-ERK1/2 was found to consistently decrease upon autophagy inhibition under CDDP stress at both 1h 6 h and also at 24h (Fig 6C and 6D). Interestingly, an increase in phospho-ERK1/2 was observed upon autophagy inhibition in OS-R cells, while JNK phosphorylation showed no alteration (data not shown). An altered MAPK signaling is probably associated with acquisition of resistance; the biological significance of the same with respect to OS-R cells is currently under investigation. However, the correlation between MAPKs, specifically JNK and autophagy observed in parental cells can specify a probable adaptive mechanism where inhibition of one survival pathway leads to the activation of another. We therefore inhibited both JNK and autophagy under CDDP stress and measured cell viability and caspase activity. A significantly decreased cell viability associated with an increase in caspase-3 activity was observed when both autophagy and JNK pathway were inhibited in CDDP-treated HOS cells (Fig 6E and 6F). Furthermore, morphological alterations also indicate the same (Fig 6G). We propose that activation of both JNK and autophagy allow HOS cells to survive drug stress. Autophagy levels however were found to be high in resistant cells too, but given that autophagy is a dynamic process, its role needs to be further delineated in resistant cells.

### Transfection of wild type p53 prevented autophagy activation upon JNK inhibition

**Fig 7:**
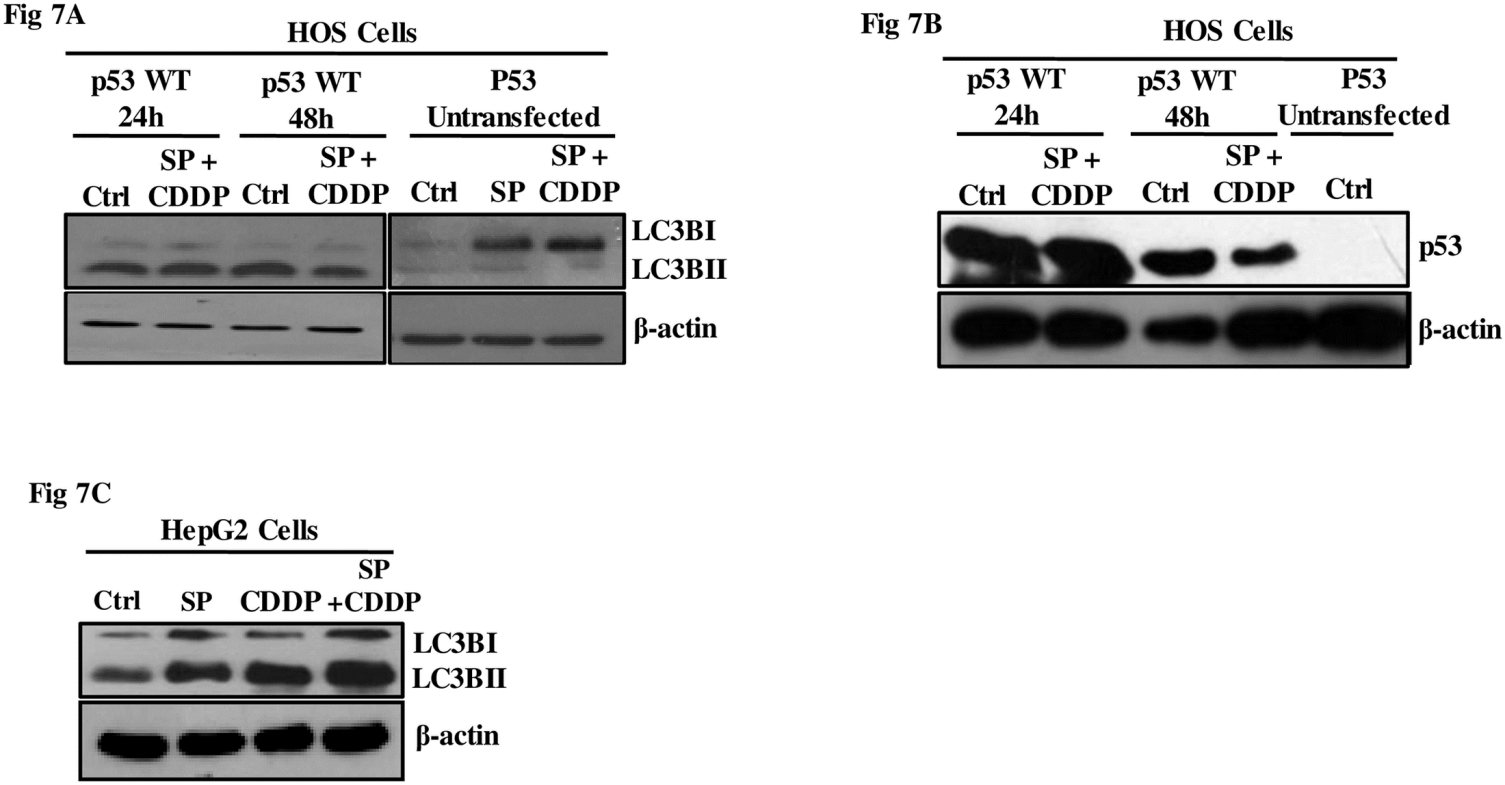
Role of p53 status in modulating autophagic response. (A) CMV-driven wild type p53 expression construct was transfected to HOS cells. Around 24h and 48h post transfection cells were exposed to specific treatments—SP (25μM) for 2h followed by CDDP (IC_50_) for 24h. Immunoblot analysis was performed to analyze LC3B-II levels. β-Actin served as a loading control and un-transfected cells served as an experimental control. (B) Transfected and un-transfected cells were analyzed for expression of p53 protein. (C) As representative of cells harboring p53 wild type protein, HepG2 cells were treated with SP (25μM) for 2h followed by CDDP (IC_50_) for 24h and harvested for analysis of LC3B by immunoblot analysis.

The HOS parental cells used in this study harbor the gain of function mutant-p53 (R156P). A plethora of existing literature functionally links JNK with wild type-p53 (wt-p53) and there are also evidences suggesting that both JNK and p53 co-operate regulating apoptosis and autophagy; however evidences are lacking in osteosarcoma. Sui et al in 2014 also reported that JNK-mediated activation of autophagy confers resistance to 5-FU in colon cancer cells [34–36]. We were hence interested to identify whether introduction of wt-p53 in HOS cells has any effect on autophagy modulation observed (an increase in LC3B was found) upon JNK inhibition. Wild-type p53 expressing vector was transfected to the HOS cells and changes were observed 24h and 48h post transfection (Fig 7A); the basal level of p53 protein stabilized in these cells under un-stimulated condition was low. Interestingly, expression of wild-type p53 in HOS cells resulted in hardly any change in protein levels of LC3B on JNK inhibition with SP (Fig 7B). The results were strikingly different from what was observed in cells un-transfected with wt-p53. Similar results were obtained when JNK signaling was inhibited in HepG2, a wild type p53 harboring cell line (Fig 7C). Also, administration of SP and CDDP did not alter the levels of LC3B significantly in HepG2 cells as was observed in HOS cells. These above results suggest that the event may be cell type specific and the presence of exogenously introduced wt-p53 can dictate upon regulation of autophagic flux upon JNK inhibition in HOS cells.

## Discussion

The underlying mechanism contributing to CDDP resistance is complex and is considered to be multi-factorial [37]. CDDP is part of the treatment regime for OS but this disease has often been resistant to conventional chemotherapies, like, CDDP. The incompletely understood mechanism associated with CDDP resistance in OS persuaded us to investigate the molecular changes contributing to drug-resistance and search for novel chemo-preventive targets that could be used in future for treatment of OS along with CDDP. A clinically relevant drug-resistant cell line (OS-R) was developed. Based on cell line information established from different patients prior and after chemotherapy we stuck to clinically relevant resistance as an approximately two-fold increase from the IC_50_ value of the parent cells [23]. Though, these cells showed low-level resistance and tend to recover in drug-free media showing relative instability in resistance features, yet these cells were preferred over high-level resistance models (with fold resistance > 10). In the high-level resistance models, though the alterations associated with resistance are massive and easier to identify, yet, they are disadvantageous as they become less relevant to the clinical setting [38]. The OS-R cells showed cross-resistance to 5-FU and over-expressed the multi-drug-resistant transporter ABCB1.

Existing reports portray that cancer cells in response to drug treatment can alter their signaling circuitry and by taking advantage of redundancy in signaling pathways maintain their survivability under drug insult [39]. There are previous reports of autophagy resisting gemcitabine and CDDP sensitivity in OS cells [15, 28], but detailed molecular studies regulating autophagy in OS cells is still poorly explored compared to other tumor models. Also, there is still a generalized debate whether, drug-induced autophagy is a protective phenomenon or is invoked to promote cell death [27]. In this study, we observed that CDDP treatment induced autophagy in HOS cells. An increase in LC3B-II protein levels, an indicator of autophagy was observed following CDDP treatment. Also, there was an increased expression of ATG-5 and ATG-12, which are known to participate in autophagosome formation and facilitate lipidation of LC3. Interestingly, inhibition of autophagy, with either CQDP or 3-MA significantly sensitized HOS cells to CDDP, proving a pro-survival role of autophagy in this context. ATG-12 has been implicated in activation of apoptosis in the literature, however, in our study, as we observed pro-survival autophagy with ATG-5 expression levels going up, we assume that ATG-12 in this context facilitates autophagy [40]. However, the OS-R cells were less responsive to CDDP-induced death upon prior autophagy inhibition though they showed higher basal autophagy levels compared to untreated HOS. Neither there was an increase in autophagic gene expression upon CDDP treatment in OS-R cells. We speculate that autophagy might play alternative functions beyond its direct link to cell survival in OS-R cells. It also indicates of mechanisms independent of autophagy to be active in OS-R cells potential enough to impart drug-resistance.

Autophagy being of utmost importance in drug-induced stress tolerance, the role of primary autophagy regulators, in this context, is crucial. Recent reports indicate the role of MAPKs like, ERK1/2 and JNK in regulating various forms of autophagy [41]. An activation of ERK1/2 signaling is known to be involved in autophagy induction upon several stimuli like, amino acid deprivation/curcumin/aurin-tricarboxylic acid etc.; while JNK participates in multiple stimulation-induced autophagic events extending from arsenic trioxide / NFκB / insulin growth factor treatment to reactive oxygen species or endoplasmic reticulum stress [42–44]. The MAPKs have also been implicated in drug-induced cyto-protective autophagy [43]. However, the role of MAPKs in regulating autophagy / apoptosis in OS is least explored. In a separate study, Hsp90 has been implicated in inducing autophagy in OS cells, but hardly any report currently explains autophagy regulation under drug stress in OS cells [45]. We observed that, in HOS cells ERK1/2 promotes apoptosis, while JNK stimulates pro-survival signals upon CDDP stress. Both ERK1/2 and JNK are implicated in literature for growth promoting or cell death inducing functions; however, we believe it is fully context and stimuli dependent. We performed a time dependent study and observed an increase in phospho-JNK level at 1, 6 and also at 24h of CDDP treatment in HOS cells. At each of these time points when phospho-JNK levels were monitored with inhibition of autophagy, we observed an increase in phospho-protein levels with respect to only CDDP treated cells. The level of phospho-JNK showed a significant increase specifically at 6h Interestingly, when we pharmacologically inhibited JNK pathway it resulted in an increased autophagic flux, while, as discussed earlier autophagy inhibition in turn caused increased JNK activation in HOS cells. JNK is reported to regulate autophagic events, but majority of the reports are biased towards autophagy activation by JNK [33]. Here, we provide evidences for crosstalk between autophagy and JNK pathway where inhibition of one pathway leads to the activation of the other. Apart from role of autophagy in cell survival under drug stress, JNK inhibition has also been implicated in reduction of osteosarcoma proliferation and differentiation. Luo *et al* in 2013 reported that bortezomib induces apoptosis and autophagy in HOS cells through regulation of ERK1/2 and JNK pathways [46]. Here, we show that both JNK and autophagy may play a pro-survival role in osteosarcoma cells after CDDP stress; and an inhibition of both JNK and autophagy caused maximal sensitivity to CDDP. However there is altered-regulation of MAPK signaling upon attainment of resistance by these cells. The role of these MAPKs controlling cell death pathways are gradually getting understood and hence is crucial to development of appropriate therapies against this fatal disease.

OS is probably one of the less investigated malignancies. Molecular studies pertaining to drug response and resistance in OS are relatively less explored, compared to other tumor models. However, survival outcomes of OS patients in clinics have not improved substantially in the last 20 years necessitating further research investigations dissecting the molecular cause to the disease and its refractoriness to chemotherapy. In this respect, since CDDP is an integral part of osteosarcoma therapy, critical insights into post CDDP response in OS, presented in this study, can have major implications in future therapeutic endeavors.

## Supporting information

S1 Fig **A**. Phase contrast images showing morphology of untreated OS-R (i), CDDP (IC50, 24h) treated OS-R cells. **B**. A fold change in caspase-3 activity in CDDP (IC_50_)-treated HOS cells and OS-R cells compared to untreated HOS and OS-R cells respectively. Activity in untreated HOS and OS-R cells was taken as "1" and a fold change in enzyme activity was calculated. **C**. Immunoblot analysis was performed to analyze expression of specific autophagic markers (ATG-5 and LC3B) following CDDP exposure (IC_50_) for 24h in OS-R cells. β-Actin served as a loading control.**D**. Immunoblot analysis was performed to analyze expression of specific proteins (PARP-1, ERK1/2 and JNK) following CDDP exposure (IC_50_) for 24h in OS-R cells. β-Actin served as a loading control.
(TIF)
