# Supplementary figures and images for: The dynamic role of autophagy and MAPK signaling in determining cell fate under cisplatin stress in osteosarcoma cells
Source: PLoS One. 2017 Jun 9;12(6):e0179203. doi: 10.1371/journal.pone.0179203 (PMC5466322; doi:10.1371/journal.pone.0179203)

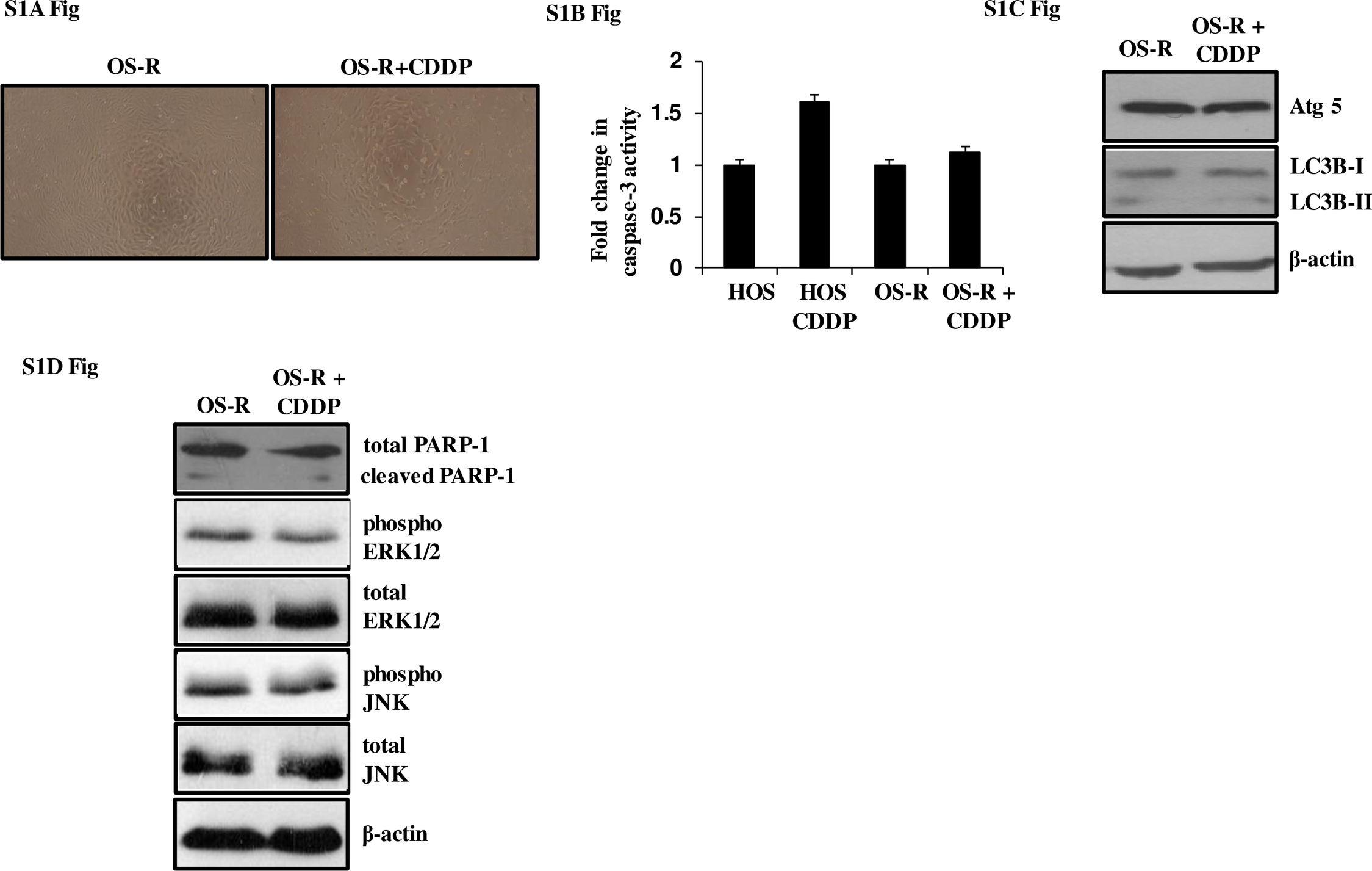

Supplement: S1 Fig — A. Phase contrast images showing morphology of untreated OS-R (i), CDDP (IC50, 24h) treated OS-R cells. B. A fold change in caspase-3 activity in CDDP (IC50)-treated HOS cells and OS-R cells compared to untreated HOS and OS-R cells respectively. Activity in untreated HOS and OS-R cells was taken as "1" and a fold change in enzyme activity was calculated. C. Immunoblot analysis was performed to analyze expression of specific autophagic markers (ATG-5 and LC3B) following CDDP exposure (IC50) for 24h in OS-R cells. β-Actin served as a loading control.D. Immunoblot analysis was performed to analyze expression of specific proteins (PARP-1, ERK1/2 and JNK) following CDDP exposure (IC50) for 24h in OS-R cells. β-Actin served as a loading control. (TIF) [file pone.0179203.s001.tif]
